# Anxiety and depression after pregnancy-related cerebral venous sinus thrombosis: associations with functional disability at discharge

**DOI:** 10.3389/fpubh.2026.1833585

**Published:** 2026-06-12

**Authors:** Ying Li, JiangShan Nie, Shuai Tao, Juan Juan Niu, Yu Tao

**Affiliations:** 1 Beijing Tiantan Hospital, Capital Medical University, Beijing, China; 2 Department of Obstetrics and Gynecology, Beijing Tiantan Hospital, Capital Medical University, Beijing, China; 3 Beijing Tiantan Hospital Department of Neurology, Beijing, China

**Keywords:** anxiety, cerebral venous sinus thrombosis, depression, functional outcome, postpartum period, pregnancy

## Abstract

**Background:**

Cerebral venous sinus thrombosis (CVST) during pregnancy and the puerperium is a rare but potentially life-threatening condition. While neurological and functional outcomes have been increasingly studied, the burden of anxiety and depression after CVST in pregnant and postpartum women remains poorly characterized.

**Methods:**

We conducted a retrospective cohort study of pregnant and postpartum women diagnosed with CVST at a tertiary referral center. Anxiety and depression were assessed at 3 months after CVST using the Hamilton Anxiety Rating Scale (HAMA) and the 24-item Hamilton Depression Rating Scale (HAMD-24). Functional outcome was evaluated using the modified Rankin Scale (mRS) at discharge. Associations between functional disability and affective symptoms were examined using linear regression models treating HAMA, HAMD-24, and mRS as continuous variables, with minimal and full adjustment for clinically relevant covariates and overlapping comorbidities, stratified by perinatal phase (antepartum vs. postpartum) with a formal mRS × phase interaction test, and supported by heteroscedasticity-consistent (HC3) robust standard errors, regression diagnostics, and rank-based Spearman correlation analyses as assumption-free sensitivity analyses.

**Results:**

Among 115 eligible patients (43 antepartum-onset, 72 postpartum-onset), symptoms of anxiety and depression were common at 3 months after CVST. The mean HAMA score was 14.9 (SD 12.0), and the mean HAMD-24 score was 15.9 (SD 12.3). Moderate-to-severe anxiety and depression were observed in 43.2 and 37.8% of patients, respectively. A worse functional outcome at discharge was strongly associated with higher anxiety and depression scores. In fully adjusted models, each one-point increase in mRS was associated with an 8.4-point increase in HAMA (95% CI 7.0–9.7) and a 7.3-point increase in HAMD-24 (95% CI 5.8–8.7, both *p* < 0.001). The dose–response relationship was directionally consistent across antepartum and postpartum subgroups (interaction *p* = 0.075 for HAMA, 0.897 for HAMD-24), and was confirmed by Spearman correlations (*ρ* = 0.75 for HAMA, 0.68 for HAMD-24; both *p* < 0.001).

**Interpretation:**

Anxiety and depression are highly prevalent following pregnancy- and puerperium-related CVST and are closely linked to functional disability at discharge. Early functional outcome may serve as a pragmatic marker to identify patients at high risk of affective disorders, highlighting the need for integrated neurological and mental health follow-up in this vulnerable population.

## Background

Cerebral venous sinus thrombosis (CVST) is a rare form of cerebrovascular disease, accounting for approximately 0.5–3% of all strokes, but it disproportionately affects young adults and women of childbearing age (1). Pregnancy and the postpartum period are well-recognized risk factors for CVST, with physiological hypercoagulability and venous stasis contributing to increased susceptibility during these states (2). Recent population-based and multicenter studies have shown that pregnancy-related CVST is associated with more severe clinical presentations, including seizures, intracranial hypertension, and impaired consciousness, and remains an essential contributor to maternal morbidity and mortality worldwide (3, 4).

Anxiety and depression are common neuropsychiatric sequelae following cerebrovascular diseases, including CVST, and may persist beyond the acute phase (5, 6). Separately, pregnancy and the postpartum period represent a vulnerable window for mental health, during which women experience a high prevalence of depression and anxiety driven by hormonal fluctuations, psychosocial stressors, and obstetric complications (7–9). Untreated perinatal anxiety and depression have been linked to adverse maternal outcomes, impaired mother–infant bonding, and long-term adverse effects on offspring neurodevelopment (8, 9).

When CVST occurs during pregnancy or the puerperium, the coexistence of an acute, potentially life-threatening neurological disorder and the physiological and psychological demands of the perinatal period may synergistically exacerbate anxiety and depressive symptoms (2, 10, 11). Concerns regarding maternal survival, fetal or neonatal safety, uncertainty about neurological prognosis, and fear of long-term disability may substantially heighten psychological distress in this population (2, 11). Moreover, neurobiological mechanisms related to cerebral venous congestion, inflammation, and brain injury may further contribute to affective disturbances, as observed in other forms of stroke (12, 13).

Despite increasing recognition of the clinical severity of pregnancy-related CVST, little is known about the burden and characteristics of anxiety and depression specifically among pregnant and postpartum women with CVST, particularly in Asian populations, where existing studies have primarily focused on clinical severity and prognosis (2, 4). Most existing studies have focused on mortality and neurological functional outcomes, with limited attention to mental health outcomes in this high-risk group (3). Therefore, the present study aimed to evaluate anxiety and depressive symptoms in pregnant and postpartum women with CVST using validated clinical scales, to provide a more comprehensive assessment of disease impact and to inform targeted multidisciplinary management strategies.

## Methods

### Setting and study design

In this retrospective cohort study, we retrospectively analyzed prospectively collected clinical data from patients with cerebral venous sinus thrombosis (CVST) treated at Beijing Tiantan Hospital, Capital Medical University, between October 2009 and December 2023. The assessments using the Hamilton Anxiety Rating Scale (HAMA) and the 24-item Hamilton Depression Rating Scale (HAMD-24) were performed solely for routine clinical management, without an initial research protocol or intent to conduct a specific study. The analysis aimed to investigate the post-CVST anxiety, depression, and quality of life state in pregnant and postpartum patients. Ethical approval for this retrospective analysis was obtained retrospectively from the Institutional Review Board (IRB) [Approval No. KY 2018-092-02], with a waiver of informed consent due to the de-identified nature of the data and minimal risk.

### Participants

Consecutive female patients treated in the study setting were screened, and those who fulfilled with the following criteria were included to the analysis: 1. age > = 18 yrs.; 2. confirmed with CVST based on clinical manifestations and neuroradiological examinations (at least one of computed tomography venography (CTV), magnetic resonance imaging (MRI) or magnetic resonance venography (MRV), or digital subtraction angiography (DSA) suggested CVST) per the Chinese Stroke Association guidelines for clinical management of cerebrovascular disorders (14); 3. onset during pregnancy or puerperium. Pregnancy was defined as the number of previous pregnancies that were clinically confirmed, by ultrasound visualization of an intrauterine gestational sac with fetus heart rate. The current pregnancy was not included in the gravidity count unless it had been previously confirmed as a clinical pregnancy. The puerperium was defined as the first 6 weeks (42 days) after delivery. Exclusion criteria were: 1. underwent surgical procedures during admission; 2. declared no permission for further scientific investigations.

### Data collection

Clinical, demographic, and outcome data were retrospectively collected from electronic medical records and institutional databases. All data were extracted by trained investigators using a standardized data collection form. Baseline demographic information included age at admission, sex, and obstetric and gynecological history, including gravidity, parity, and menstrual regularity. Pre-existing medical comorbidities were recorded based on documented diagnoses, including autoimmune disease, cardiovascular disease, dyslipidemia, anemia, thyroid disorders, and other chronic systemic conditions. Neurological status before and at presentation was assessed using the GCS and mRS. Clinical manifestations during the acute phase were systematically recorded, including headache, nausea or vomiting, visual disturbance or papilledema, seizures, focal neurological deficits, cognitive impairment, and other neurological symptoms. Radiological involvement of cerebral venous structures was documented based on neuroimaging reports, including the number and distribution of affected venous sinuses. CVST-related complications and comorbid conditions were extracted, including cerebral infarction, intracranial hemorrhage, liver function abnormalities, prothrombotic conditions, cerebral herniation, systemic embolism, and intercurrent infections. Acute and secondary treatments during hospitalization were recorded, including anticoagulation strategies (warfarin, low-molecular-weight heparin, or low-dose heparin), antiplatelet therapy, and venous sinus stenting. Functional outcome at discharge was assessed using mRS. At 3 months after CVST onset, affective symptoms were evaluated using the HAMA and the HAMD-24.

### Assessments and definitions

Several standardized scales were used in the analysis to ensure objective evaluation. Scales were recategorized according to established cutoff values. Level of consciousness was assessed using the GCS and categorized as mild (13–15), moderate (9–12), or severe (≤8). Neurological functional outcome was assessed using the mRS, with scores ranging from 0 (no symptoms) to 6 (death). Outcomes were dichotomized as good (mRS 0–2) and poor (mRS 3–6). Anxiety severity was assessed using the HAMA, with total scores interpreted as no or minimal anxiety (0–7), mild anxiety (8–14), moderate anxiety (15–23), and severe anxiety (≥24). Depressive symptoms were assessed using the HAMD-24, with total scores categorized as no depression (0–7), mild depression (8–16), moderate depression (17–23), and severe depression (≥24). Detailed scales were presented in Supplementary Tables S1–S4. Regular menstruation was defined as cyclic uterine bleeding that occurs at predictable intervals, meeting all of the following four criteria: (1) The interval from the first day of one menstrual period to the first day of the next is consistently between 21 and 35 days; (2) The variation in length between consecutive cycles is less than or equal to 7–9 days. (3) The menstrual bleeding itself lasts for 2–8 days. (4) The total blood loss per cycle is typically between 20 and 60 milliliters, which is not associated with excessive clotting or severe pain that disrupts regular activity, and does not require abnormally frequent pad/tampon changes. Prothrombotic conditions were defined as persistent hematological disorders identified by specific laboratory abnormalities in the coagulation system that confer an increased lifelong risk of thrombosis. Anemia was defined as a hemoglobin concentration below 110 g/L.

### Statistical analysis

Continuous variables were summarized as mean ± standard deviation (SD) or median with interquartile range (IQR), as appropriate based on distributional characteristics. Categorical variables were described as counts and percentages. Anxiety and depressive symptoms at 3 months after CVST were assessed using the HAMA and HAMD-24, respectively, and were analyzed as continuous outcomes. Functional outcome was evaluated using the modified Rankin Scale (mRS) at discharge.

Primary associations between mRS and affective symptom scores were examined using linear regression models. Unadjusted models included mRS as the sole predictor. Minimally adjusted models additionally included age, GCS category at admission, and CVST-related stroke (ischemic and/or hemorrhagic) as covariates selected *a priori* based on clinical relevance and prior literature. A directed acyclic graph (DAG) was constructed to illustrate the hypothesized relationships among functional disability, affective symptoms, and relevant clinical variables. Although mRS is an ordinal scale, it was modelled as a continuous predictor shifted by 1 to estimate overall functional disability gradients across the full outcome spectrum and to preserve information across adjacent score levels. In contrast, the Glasgow Coma Scale (GCS) at admission was analyzed as a categorical variable, reflecting established clinical severity thresholds and its non-linear relationship with neurological status in the acute phase. To assess the robustness of the observed associations without assuming linearity or normality, rank-based Spearman correlation analyses were performed as sensitivity analyses.

To examine whether the disability–affective symptom relationship differed by perinatal phase, we conducted stratified linear regression with a formal interaction term (mRS × perinatal phase) within a single model adjusted for age, GCS category, and any cerebral hemorrhage and/or infarction. We further fit a fully adjusted model that additionally included perinatal phase, any autoimmune disease, and anemia as covariates to address potential confounding by overlapping comorbidities. To evaluate whether the association reflected obstetric recovery rather than neurological injury, we performed a sensitivity analysis within the antepartum subgroup that adjusted additionally for obstetric outcome (artificial abortion, mid-trimester induction, or live birth). Heteroscedasticity-consistent (HC3) robust standard errors were computed for all linear-model inference, and residuals were assessed for heteroscedasticity (Breusch–Pagan test) and normality (Shapiro–Wilk test) to verify modelling assumptions. Rank-based Spearman correlations between discharge mRS and 3-month HAMA and HAMD-24 scores were calculated overall and within each perinatal subgroup as assumption-free sensitivity analyses. During preparation of this revision, we also re-verified all HAMA and HAMD-24 entries against the source clinical records and re-extracted obstetric variables (perinatal phase, gestational week at CVST onset, and obstetric outcome) that were not included in the original analytic dataset; minor data-entry corrections to a small number of values were applied during this audit and are reflected in the revised Results, Tables 1, 2, and Supplementary Tables S5–S9.

**Table 1:** Baseline characteristics of the study population.

Characteristic	Overall (*N* = 115)	Antepartum (*n* = 43)	Postpartum (*n* = 72)	*p-*value
Demographics				
Age (years)*	37.1 ± 9.9; 40.0 [30.0, 45.0]	26.1 ± 5.9; 27.0 [21.0, 31.0]	43.6 ± 4.6; 43.5 [40.0, 47.0]	<0.001
Female	115 (100)	43 (100)	72 (100)	—
Perinatal characteristics				
Antepartum onset	43 (37.4)	43 (100)	—	—
First trimester (<12 wk)	26 (22.6)	26 (60.5)	—	—
Second trimester (12–27 wk)	11 (9.6)	11 (25.5)	—	—
Third trimester (≥28 wk)	6 (5.2)	6 (14.0)	—	—
Postpartum onset	72 (62.6)	—	72 (100)	—
Early puerperium (<7 d)‡	17 (14.8)	—	17 (23.6)	—
Late puerperium (7–42 d)‡	55 (47.8)	—	55 (76.4)	—
Obstetric outcome at follow-up†				
Artificial abortion	28 (24.3)	28 (65.1)	—	—
Mid-trimester induction	9 (7.8)	9 (20.9)	—	—
Live birth	6 (5.2)	6 (14.0)	—	—
Already delivered before CVST	72 (62.6)	—	72 (100)	—
Pregnancy resolved by 3-mo FU	115 (100)	43 (100)	72 (100)	—
Obstetric and gynecological history				
Gravidity§				<0.001
0	24 (20.9)	22 (51.2)	2 (2.8)	
1	50 (43.5)	16 (37.2)	34 (47.2)	
2	27 (23.5)	3 (7.0)	24 (33.3)	
≥3	14 (12.2)	2 (4.7)	12 (16.7)	
Parity				<0.001
0	39 (33.9)	26 (60.5)	13 (18.1)	
1	54 (47.0)	16 (37.2)	38 (52.8)	
2	19 (16.5)	0 (0)	19 (26.4)	
3	3 (2.6)	1 (2.3)	2 (2.8)	
Regular menstruation	98 (85.2)	33 (76.7)	65 (90.3)	0.048
Comorbidities				
Autoimmune disease	57 (49.6)	34 (79.1)	23 (31.9)	<0.001
Anemia	42 (36.5)	14 (32.6)	28 (38.9)	0.495
Hyperlipidemia	34 (29.6)	9 (20.9)	25 (34.7)	0.117
Cardiovascular disease	19 (16.5)	5 (11.6)	14 (19.4)	0.275
Thyroid disorder	5 (4.3)	2 (4.7)	3 (4.2)	>0.999
Prior depression¶	2 (1.7)	2 (4.7)	0 (0)	0.138

*Gravidity refers to the number of previously confirmed clinical pregnancies (see Methods for definition). Patients presenting in early first trimester without confirmed intrauterine pregnancy at data entry may be recorded as Gravidity = 0. †Autoimmune diseases included systemic rheumatic diseases and other organ-specific autoimmune conditions. ‡Cardiovascular disease included hypertension, coronary artery disease, and other documented cardiovascular conditions. §Psychiatric disorders referred to a previously documented diagnosis of depression prior to admission. IQR, interquartile range.

**Table 2:** Clinical characteristics, CVST involvement, complications, and treatments.

**Characteristic**	**Overall** **(*N* = 115)**	**Antepartum** **(*n* = 43)**	**Postpartum** **(*n* = 72)**	***p-*value**
Hospitalization metrics				
Length of stay (days)*	17.0 ± 10.7; 15.0 [8.0, 21.0]	16.9 ± 8.7; 16.0 [13.0, 21.0]	17.1 ± 11.8; 15.0 [8.0, 21.5]	0.575
Intracranial pressure (mmH₂O)*	272 ± 81; 260 [235, 330]	278 ± 76; 280 [235, 350]	268 ± 84; 255 [232, 330]	0.551
Functional status on admission				
Glasgow Coma Scale score				0.194
13–15 (mild impairment)	101 (87.8)	40 (93.0)	61 (84.7)	
9–12 (moderate impairment)	5 (4.3)	0 (0)	5 (6.9)	
≤8 (severe impairment)	9 (7.8)	3 (7.0)	6 (8.3)	
Pre-CVST modified Rankin Scale				0.755
0–2 (good functional status)	90 (78.3)	33 (76.7)	57 (79.2)	
3–6 (poor functional status)	25 (21.7)	10 (23.3)	15 (20.8)	
Neurological manifestations at presentation				
Headache	93 (80.9)	38 (88.4)	55 (76.4)	0.114
Nausea	97 (84.3)	37 (86.0)	60 (83.3)	0.698
Vomiting	101 (87.8)	40 (93.0)	61 (84.7)	0.246
Blurred vision or papilloedema	58 (50.4)	23 (53.5)	35 (48.6)	0.613
Convulsions	20 (17.4)	9 (20.9)	11 (15.3)	0.439
Hemiparesis	18 (15.7)	5 (11.6)	13 (18.1)	0.359
Neck rigidity	14 (12.2)	3 (7.0)	11 (15.3)	0.246
Sensory disturbance	14 (12.2)	11 (25.6)	3 (4.2)	0.002
Aphasia	13 (11.3)	4 (9.3)	9 (12.5)	0.764
Dysphagia	12 (10.4)	9 (20.9)	3 (4.2)	0.009
Tinnitus	11 (9.6)	4 (9.3)	7 (9.7)	>0.999
Cognitive impairment	8 (7.0)	1 (2.3)	7 (9.7)	0.255
Diplopia	7 (6.1)	5 (11.6)	2 (2.8)	0.101
Facial paralysis	1 (0.9)	0 (0)	1 (1.4)	>0.999
Dysarthria	1 (0.9)	0 (0)	1 (1.4)	>0.999
CVST anatomical involvement†				
No discernible sinus thrombosis	2 (1.7)	1 (2.3)	1 (1.4)	>0.999
Single sinus	27 (23.5)	12 (27.9)	15 (20.8)	0.387
Two sinuses	48 (41.7)	18 (41.9)	30 (41.7)	0.984
Three or more sinuses	38 (33.0)	12 (27.9)	26 (36.1)	0.365
Multisinus involvement (≥2 sinuses)	86 (74.8)	30 (69.8)	56 (77.8)	0.339
CVST-related complications and associated conditions				
Cerebral infarction	27 (23.5)	10 (23.3)	17 (23.6)	0.965
Intracranial hemorrhage	20 (17.4)	7 (16.3)	13 (18.1)	0.808
Hemorrhage and/or infarction	39 (33.9)	13 (30.2)	26 (36.1)	0.519
Liver function abnormality	17 (14.8)	7 (16.3)	10 (13.9)	0.727
Prothrombotic conditions	16 (13.9)	5 (11.6)	11 (15.3)	0.584
Cerebral herniation	10 (8.7)	3 (7.0)	7 (9.7)	0.741
Systemic embolism	9 (7.8)	1 (2.3)	8 (11.1)	0.150
Upper respiratory tract infection	5 (4.3)	2 (4.7)	3 (4.2)	>0.999
Treatments during hospitalization				
Warfarin	105 (91.3)	41 (95.3)	64 (88.9)	0.317
Low-molecular-weight heparin	73 (63.5)	27 (62.8)	46 (63.9)	0.906
Dual antiplatelet therapy	36 (31.3)	10 (23.3)	26 (36.1)	0.150
Low-dose unfractionated heparin	25 (21.7)	11 (25.6)	14 (19.4)	0.440
Venous sinus stenting	24 (20.9)	8 (18.6)	16 (22.2)	0.644
Functional outcome at discharge				
Modified Rankin Scale at discharge				0.269
0–2 (good functional outcome)	99 (86.1)	39 (90.7)	60 (83.3)	
3–6 (poor functional outcome)	16 (13.9)	4 (9.3)	12 (16.7)	
Affective outcomes at 3-month follow-up‡				
HAMA score (0–56)*	14.9 ± 12.0; 13.0 [5.0, 21.0]	15.3 ± 10.3; 14.5 [8.0, 20.0]	14.7 ± 13.0; 12.0 [4.0, 21.0]	0.394
HAMA moderate-to-severe (≥15)	48 (43.2)	21 (50.0)	27 (39.1)	0.233
HAMD-24 score (0–76)*	15.9 ± 12.3; 15.0 [8.0, 21.0]	16.7 ± 11.5; 15.0 [9.0, 21.0]	15.5 ± 12.8; 14.0 [5.0, 20.0]	0.338
HAMD-24 moderate-to-severe (≥17)	42 (37.8)	16 (38.1)	26 (37.7)	0.906

*Continuous variables are shown as mean ± SD; median [IQR]. Categorical variables are shown as *n* (%). Percentages may not total 100 because of rounding. *p*-values compare antepartum vs postpartum subgroups (continuous: Wilcoxon rank-sum; categorical: Pearson χ^2^ or Fisher exact, as appropriate). †Anatomical involvement is summarized by the number of sinuses involved on neuroimaging, replacing the pattern-based categorization used in earlier versions for greater interpretability. The most common patterns were transverse + sigmoid, and transverse + sigmoid + superior sagittal. ‡Sample size for the affective outcomes is 111 because four patients died before the 3-month assessment (1 antepartum, 3 postpartum). HAMA = Hamilton Anxiety Rating Scale; HAMD-24 = 24-item Hamilton Depression Rating Scale. *Other patterns included Great cerebral vein, Straight sinus, Internal jugular vein.

All analyses were conducted using R software (version 4.5.2; R Foundation for Statistical Computing, Vienna, Austria). A two-sided *p-*value <0.05 was considered statistically significant.

## Results

Of 131 pregnant or postpartum women with CVST screened during the study period, 15 underwent surgical intervention during admission and 1 declined participation; the final analytic cohort comprised 115 women, of whom 43 (37.4%) had antepartum-onset and 72 (62.6%) had postpartum-onset CVST. Four patients (1 antepartum, 3 postpartum) died within 3 months of CVST and were not included in the affective-symptom analyses, leaving 111 patients with HAMA and HAMD-24 measurements available at follow-up. All 115 patients had ended the index pregnancy by the 3-month assessment, with no ongoing pregnancies.

Baseline demographic and clinical characteristics of the study population are summarized in Table 1. The median age was 40.0 years (IQR 30.0–45.0), and all included patients were female. Among the antepartum cases, the distribution was as follows: 26 (60.5%) occurred in the first trimester, 11 (25.5%) in the second trimester, and 6 (14.0%) in the third trimester. Within the puerperium group, the majority of cases were diagnosed during the late puerperium (7–42 days, 55 patients, 76.4%), while 17 patients (23.6%) were diagnosed in the early puerperium (<7 days). A substantial proportion had a history of pregnancy, with gravidity ≥1 reported in the majority of patients, and regular menstruation was observed in more than four-fifths of the cohort. With respect to systemic medical history, autoimmune diseases were common, followed by anemia and hyperlipidemia, whereas thyroid disorders, rheumatic diseases, and prior depression were infrequent. Other comorbid conditions were reported in one-fifth of patients. Antepartum-onset patients were more frequently nulliparous (60.5% vs. 18.1%; *p* < 0.001) and showed a markedly higher prevalence of autoimmune disease (79.1% vs. 31.9%; *p* < 0.001), whereas the prevalence of anemia, hyperlipidemia, cardiovascular disease, and thyroid disorder did not differ significantly between subgroups (all *p* ≥ 0.117). A previously documented diagnosis of depression was rare and confined to the antepartum subgroup (2/43 [4.7%] vs. 0/72 [0%]; Fisher exact *p* = 0.138).

Clinical status on admission and neurological manifestations, patterns of venous sinus involvement, CVST-related complications and treatment strategies are detailed in Table 2. A total of 101 patients (87.8%) had a Glasgow Coma Scale score of 13–15 at admission, moderate or severe impairment (5,4.3%;9,7.8%) was observed in a minority. Before CVST onset, 90 patients (78.3%) had a modified Rankin Scale score of 0–2, 25 patients (21.7%) scored 3–6. Headache (93 patients, 80.9%) and nausea or vomiting (101 patients, 87.8%) were the most frequent presenting symptoms, followed by blurred vision or papilledema (58 patients, 50.4%). Seizures and focal neurological deficits, including hemiparesis (18 patients, 15.7%) and aphasia (13 patients, 11.3%), were less common. Other neurological manifestations occurred in a smaller proportion of patients. The anatomical distribution of the thrombus involved a single sinus in 31 patients (27.0%), while 84 patients (73.0%) had involvement of multiple sinuses or veins. The most common pattern was isolated involvement of the transverse sinus and sigmoid sinus in 38 patients (33.0%), followed by involvement of the transverse sinus, sigmoid sinus, and superior sagittal sinus in 16 patients (13.9%). Other patterns included isolated transverse sinus involvement in 12 patients (10.4%), isolated sigmoid sinus in 7 patients (6.1%), and transverse sinus and superior sagittal sinus involvement in 6 patients (5.2%). Isolated superior sagittal sinus involvement was also observed in 6 patients (5.2%). Less frequent patterns included involvement of the transverse sinus, sigmoid sinus, and internal jugular vein in 4 patients (3.5%), and isolated involvement of the superficial cerebral veins in 2 patients (1.7%). Regarding complications and associated conditions identified during hospitalization, cerebral infarction and intracranial hemorrhage (47 patients, 40.9%) were the most frequently observed events. Prothrombotic conditions (16 patients, 13.9%) and liver function (17 patients, 14.8%) abnormalities were also commonly identified. Cerebral herniation (10 patients, 8.7%) and systemic embolism (9 patients, 7.8%) occurred in a minority of patients, while upper respiratory tract infection (5 patients, 4.3%) and other complications (7 patients, 6.1%) were infrequent. Anticoagulation therapy was widely used, with warfarin (105 patients, 91.3%) and low-molecular-weight heparin (73 patients, 63.5%) being the most frequently prescribed agents. Dual antiplatelet therapy (36 patients, 31.1%) and low-dose unfractionated heparin (25 patients, 21.7%) were used in a subset of patients. Endovascular treatment with venous sinus stenting was performed in approximately one-fifth of the cohort (24 patients, 20.9%).

**Figure 1:**
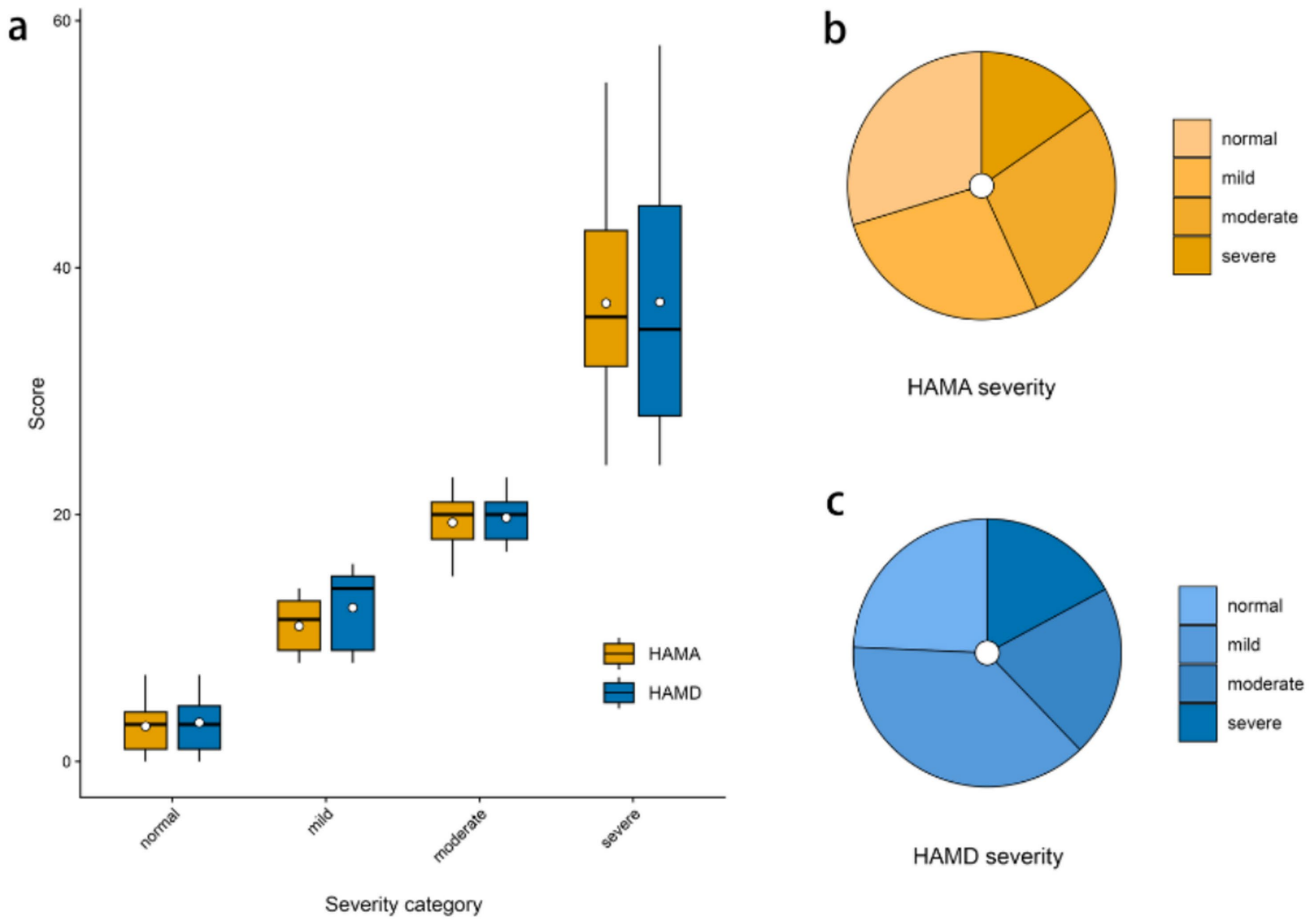
Distribution of anxiety and depression severity. **(a)** Boxplots showing Hamilton Anxiety Rating Scale (HAMA) and Hamilton Depression Rating Scale (HAMD-24) scores stratified by the corresponding scale’s own severity categories (normal, mild, moderate, and severe), defined according to established cut-offs (HAMA: 0–7, 8–14, 15–23, ≥24; HAMD-24: 0–7, 8–16, 17–23, ≥24). Boxes represent the interquartile range (IQR), horizontal lines indicate the median, whiskers denote the range excluding outliers, and white dots indicate the mean value. HAMA and HAMD-24 scores are displayed side by side within each severity category. **(b)** Pie chart illustrating the distribution of HAMA severity categories in the study population. **(c)** Pie chart illustrating the distribution of HAMD-24 severity categories in the study population. Severity categories for HAMA and HAMD-24 were defined according to established cut-offs. Percentages represent the proportion of patients within each severity category.

At the 3-month follow-up, the mean HAMA score was 14.9 (SD 12.0; median 13.0, IQR 5.0–21.0) and the mean HAMD-24 score was 15.9 (SD 12.3; median 15.0, IQR 8.0–21.0). Using established severity thresholds, 33 patients (29.7%) had no or minimal anxiety, 30 (27.0%) had mild anxiety, 31 (27.9%) had moderate anxiety, and 17 (15.3%) had severe anxiety; for HAMD-24, 27 (24.3%), 42 (37.8%), 23 (20.7%), and 19 (17.1%) fell into the corresponding categories (Figure 1). Mean affective scores did not differ significantly between antepartum and postpartum subgroups (HAMA 15.3 vs. 14.7, *p* = 0.394; HAMD-24 16.7 vs. 15.5, *p* = 0.338), nor did the proportion of patients with moderate-to-severe anxiety (50.0% vs. 39.1%; *p* = 0.233) or moderate-to-severe depression (38.1% vs. 37.7%; *p* = 0.906).

**Figure 2:**
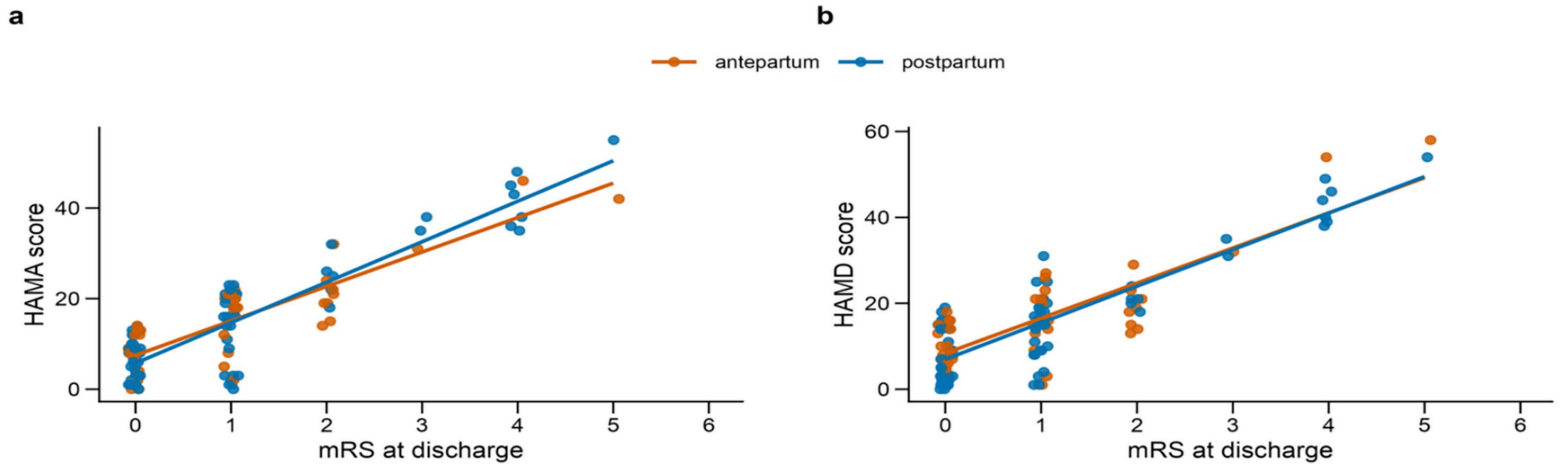
Association between functional outcome at discharge and 3-month affective symptoms, stratified by perinatal phase. **(a)** Scatter plot of modified Rankin Scale (mRS) score at discharge versus Hamilton Anxiety Rating Scale (HAMA) score. **(b)** Scatter plot of mRS at discharge versus 24-item Hamilton Depression Rating Scale (HAMD-24) score. Each dot represents one patient, color-coded by perinatal phase (orange = antepartum, *n* = 43; blue = postpartum, *n* = 72). Solid lines indicate phase-specific fitted linear regressions. Higher mRS scores indicate worse functional outcome; higher HAMA and HAMD-24 scores indicate greater symptom severity.

Greater functional disability at discharge and 3-month affective symptoms. Greater functional disability at discharge was strongly associated with higher 3-month affective symptom severity (Figure 2 and Supplementary Figure S2). In unadjusted linear regression, each one-point increase in mRS at discharge was associated with an 8.52-point increase in HAMA score (95% CI 7.61–9.42) and an 8.41-point increase in HAMD-24 score (95% CI 7.39–9.42), both *p* < 0.001. After minimal adjustment for age, GCS category at admission, and any cerebral hemorrhage and/or infarction, each one-point increase in mRS remained associated with an 8.45-point increase in HAMA (95% CI 7.13–9.76) and a 7.42-point increase in HAMD-24 (95% CI 5.97–8.86), both p < 0.001. The full coefficient table is provided in Supplementary Table S5.

**Figure 3:**
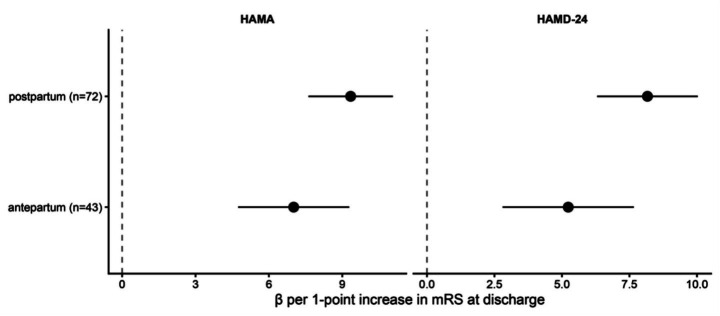
Forest plot of stratified *β* estimates by perinatal phase. Point estimates and 95% confidence intervals for the regression coefficient of modified Rankin Scale (mRS) score at discharge, fitted separately within antepartum-onset (*n* = 43) and postpartum-onset (*n* = 72) subgroups, with the same minimally adjusted covariate set (age, GCS category at admission, and any cerebral haemorrhage and/or infarction). Each panel shows one outcome (HAMA on the left; HAMD-24 on the right). The vertical dashed line at zero indicates no association. Higher mRS scores indicate worse functional outcome; higher HAMA and HAMD-24 scores indicate greater symptom severity.

To assess whether the disability–affective relationship differed by perinatal phase, we performed pre-specified stratified linear regression with a formal interaction test (Figure 3 and Supplementary Table S6). Within antepartum-onset patients (*n* = 43), each one-point increase in discharge mRS was associated with a 7.01-point rise in HAMA (95% CI 4.75–9.28) and a 5.23-point rise in HAMD-24 (95% CI 2.81–7.66); within postpartum-onset patients (*n* = 72), the corresponding estimates were larger in magnitude (HAMA *β* = 9.34, 95% CI 7.62–11.06; HAMD-24 *β* = 8.17, 95% CI 6.31–10.02); all four estimates *p* < 0.001. The mRS × perinatal-phase interaction term did not reach statistical significance for either outcome (HAMA *p* = 0.075; HAMD-24 *p* = 0.897), indicating that the dose–response relationship was directionally consistent across subgroups despite a numerically steeper slope in the postpartum subgroup. Stratified Spearman correlations were concordant (antepartum *ρ* = 0.779 for HAMA and 0.626 for HAMD-24; postpartum ρ = 0.712 for HAMA and 0.699 for HAMD-24; all *p* < 0.001; Supplementary Table S8).

Sensitivity analyses. Several pre-specified sensitivity analyses supported the primary findings (Supplementary Tables S5, S7, S9). First, fully adjusted models that additionally included perinatal phase, any autoimmune disease, and anemia produced effect estimates essentially unchanged from the minimally adjusted models (HAMA *β* = 8.37, 95% CI 7.02–9.72; HAMD-24 *β* = 7.25, 95% CI 5.78–8.72; both *p* < 0.001), arguing against substantial confounding by these comorbidity profiles. Second, within the antepartum subgroup, additional adjustment for obstetric outcome (artificial abortion, mid-trimester induction, or live birth) left the mRS coefficient virtually unchanged (HAMA *β* = 6.97, 95% CI 4.62–9.32; HAMD-24 *β* = 5.19, 95% CI 2.67–7.71; both *p* < 0.001), and none of the obstetric-outcome contrasts approached statistical significance (all *p* ≥ 0.687), indicating that the observed disability–affect association did not appear to reflect the mode of pregnancy resolution. Third, heteroscedasticity-consistent (HC3) robust standard errors yielded confidence intervals nearly identical to the model-based estimates. Diagnostic testing showed no evidence of heteroscedasticity in either fully adjusted model (Breusch–Pagan *p* = 0.326 for HAMA, *p* = 0.488 for HAMD-24); HAMA residuals showed mild deviation from normality (Shapiro–Wilk *p* = 0.002), but the assumption-free overall Spearman analyses yielded *ρ* = 0.752 for HAMA and ρ = 0.680 for HAMD-24 (both *p* < 0.001; Supplementary Figure S1), confirming the same conclusion. Together, these analyses indicate that the dose–response relationship between functional disability at discharge and 3-month affective symptoms is robust to choice of adjustment set, distributional assumptions, and the obstetric pathway by which the index pregnancy was resolved.

## Discussion

In this retrospective cohort of women with pregnancy- or peripartum-related CVST, anxiety and depressive symptoms were frequent at 3 months and increased in parallel with functional disability at discharge. To our knowledge, the mental health sequelae of pregnancy- and puerperium-associated CVST have rarely been quantified using standardized clinician-rated scales, despite pregnancy and the puerperium being recognized risk periods for CVST (15). Our findings extend the CVST outcome literature by systematically characterizing post-event anxiety and depression in a population that is both clinically vulnerable and historically under-represented in neuropsychiatric outcome studies.

Beyond acute survival and neurological stabilization, the long-term recovery of critically ill obstetric patients has been increasingly emphasized in contemporary maternal health care frameworks in China (15–17). Women affected by severe pregnancy- or puerperium-related conditions, such as CVST, are typically enrolled in structured post-discharge follow-up in the study center. These programs aim to monitor not only overall recovery and functional status, but also broader physical and psychological wellbeing, including anxiety and depression assessment, during the postpartum period. As a result, the analyses of HAMA and HAMD-24 scores at 3 months after CVST were feasible within the context. Perinatal mood and anxiety disorders are common and clinically consequential. Large-scale evidence indicates that depressive symptoms during pregnancy and the postpartum period are prevalent and are associated with substantial impairment in maternal functioning, bonding, and broader family wellbeing (18). Against this background, neurovascular events such as CVST may add biological stress (inflammation, endocrine perturbation, pain, sleep disruption) and psychosocial stress (infant care demands, role transition), potentially amplifying vulnerability to anxiety and depression in the months following the acute illness. Although CVST is often considered a stroke subtype with favorable survival, accumulating work suggests that many survivors experience persistent sequelae—fatigue, cognitive complaints, reduced participation, and impaired quality of life (19). These long-term burdens plausibly intersect with mood symptoms, yet dedicated psychiatric outcome reporting remains limited in CVST, and is even less frequently focused on pregnancy- or puerperium-associated cases. Prior work also supports a close relationship between functional disability and mood outcomes after stroke, and early depressive symptoms can predict worse, longer-term quality of life (20). Our observation that worse mRS at discharge tracked with higher HAMA and HAMD-24 scores is consistent with this disability–mood linkage, suggesting that functional outcome may be a pragmatic anchor for early mental health surveillance after CVST.

To account for potential confounding and to determine whether this association was independent of other clinical factors, we further examined the relationship between functional disability and affective symptoms using adjusted regression models. Covariate selection was guided by established clinical relevance and prior literature. Age was included as a broad determinant of stroke recovery, psychosocial resources, and baseline vulnerability to affective disorders, and has been frequently examined in studies of post-stroke anxiety and depression (21). Neurological severity at presentation was captured using the Glasgow Coma Scale category as a proxy for acute illness severity, a construct consistently associated with subsequent depressive symptoms and psychological outcomes after stroke (21). Stroke was included as a covariate because it is a well-recognized precipitating factor for neuropsychiatric sequelae (12).

During the analysis, HAMA and HAMD-24 scores were modelled as continuous outcomes using linear regression to examine the association between functional outcomes and affective symptoms. Although the modified Rankin Scale (mRS) is an ordinal measure, it was modelled as a continuous predictor to estimate overall gradients of functional disability across its full range, an approach commonly adopted in stroke research when the aim is to characterize dose–response relationships rather than discrete outcome states (22). Treating validated symptom scales as continuous variables preserves information across the full distribution and maximizes statistical efficiency. In contrast, dichotomizing or ordinalizing symptom scores—such as applying logistic or ordinal regression to severity categories—has been shown to reduce statistical power, obscure within-category variability, and yield estimates that depend on arbitrary cut-points, potentially resulting in biased or misleading inferences (23–25). Linear regression provides effect estimates that are directly interpretable in clinical terms, expressed as the mean change in anxiety or depressive symptom scores per one-point increase in functional disability. To assess the robustness of these findings without imposing linearity or distributional assumptions, rank-based Spearman correlation analyses were performed as an assumption-free sensitivity analysis, demonstrating concordant association directions and supporting the stability of the primary modelling approach.

The dose–response relationship between functional disability at discharge and 3-month affective symptoms was directionally consistent across antepartum and postpartum subgroups, with no statistically significant heterogeneity (interaction *p* = 0.075 for HAMA and 0.897 for HAMD-24). The numerically steeper slope observed in the postpartum subgroup (*β* = 9.34 for HAMA; *β* = 8.17 for HAMD-24) is in keeping with the hypothesis that the puerperium amplifies vulnerability to mood disturbance through additional biological and psychosocial stressors, including hormonal withdrawal, sleep disruption, lactation-related fatigue, and the cumulative demands of infant care. Within the antepartum subgroup, the mRS coefficient remained essentially unchanged after adjustment for obstetric outcome (artificial abortion, mid-trimester induction, or live birth; HAMA *β* = 6.97 vs. 7.01; HAMD-24 *β* = 5.19 vs. 5.23), arguing against the alternative interpretation that functional impairment at discharge primarily reflected obstetric recovery rather than neurological injury.

In considering the complex causal landscape of pregnancy- or puerperium-related CVST, where neurological injury, perinatal physiological adaptation, and a substantial burden of comorbidities act simultaneously, we conducted layered sensitivity analyses to triangulate the disability–affect association. The fully adjusted model, which additionally included perinatal phase, autoimmune disease, and anemia, produced effect estimates that were nearly identical to those of the minimally adjusted model (HAMA *β* = 8.37 vs. 8.45; HAMD-24 *β* = 7.25 vs. 7.42), suggesting that the observed dose–response relationship is not substantially confounded by these comorbidity profiles. Heteroscedasticity-consistent (HC3) standard errors and rank-based Spearman correlations yielded the same conclusion despite mild residual non-normality in the HAMA model (Shapiro–Wilk *p* = 0.002). Together, these analyses indicate that the association between functional disability at discharge and 3-month anxiety and depression severity is robust to the choice of adjustment set, distributional assumptions, and the obstetric pathway by which the index pregnancy was resolved.

Clinically, our findings support the integration of mental health screening into post-CVST follow-up, particularly among women discharged with higher mRS scores, in whom psychological morbidity may compound functional recovery. A pragmatic care pathway may include early identification of high-risk patients based on discharge mRS and acute severity markers, followed by structured assessment of anxiety and depression at the 3-month follow-up using validated instruments, and stepped-care referral to psychological or psychiatric services when moderate-to-severe symptoms are identified. Future studies may evaluate whether early identification and targeted intervention for post-CVST psychological symptoms can improve functional and quality-of-life outcomes in this vulnerable population. These clinical implications should be regarded as preliminary and require validation in prospective and comparative studies before routine adoption.

This study has several limitations. First, the retrospective design introduces potential selection and information biases, and unmeasured confounding cannot be entirely excluded. Second, the sample size was modest (*n* = 115), reflecting the low incidence of CVST and the even rarer pregnancy- or puerperium-associated subset, which may limit precision for multivariable estimates and subgroup inference (15). Owing to the sample size and data structure, we did not undertake predictive modelling, as such models may yield unstable estimates and limited interpretability under these conditions. Third, we lacked systematic baseline (pre-CVST) HAMA/HAMD-24 assessments. This is particularly relevant because a small number of participants had a documented history of depression. Without pre-event symptom quantification, we cannot fully disentangle new-onset versus pre-existing affective symptoms. However, acute-phase illness effects (pain, sleep disruption, neurological deficits) may inflate symptom scores. The observed associations should therefore be interpreted as correlational rather than causal. Fourth, our cohort lacked a contemporaneous comparison group of pregnant or postpartum women without CVST. Because the background prevalence of clinically significant anxiety or depression during the perinatal period is itself substantial, some of the affective symptom burden observed at 3 months may reflect underlying perinatal vulnerability rather than CVST-specific neuropsychiatric sequelae. Our dose–response findings should therefore be interpreted as internal to a CVST cohort: they characterize how functional disability tracks affective symptom severity within affected women, but do not establish that CVST itself elevates the risk of affective disorders above the perinatal baseline. Comparative studies including matched perinatal women without CVST will be required to address that question. Fifth, affective symptoms were measured at a single 3-month time point. We therefore cannot distinguish transient adjustment reactions from persistent neuropsychiatric morbidity, nor characterize the trajectory of recovery. Longitudinal repeated assessments would be required to do so.

## Conclusion

In this cohort of pregnant and postpartum women with cerebral venous sinus thrombosis, anxiety and depression were frequent at 3 months after the acute event and showed a strong association with functional disability at discharge. These findings underscore the substantial mental health burden accompanying CVST beyond neurological recovery. Functional outcome, as reflected by the modified Rankin Scale, may provide a simple and clinically meaningful anchor for early identification of patients at heightened risk of affective symptoms. Incorporating routine mental health surveillance into post-CVST care pathways for critically ill obstetric patients may help address an under-recognized dimension of recovery and improve longer-term outcomes. Whether such surveillance also improves long-term clinical outcomes will require prospective comparative studies; the present analyses establish only an internal dose–response relationship within women already affected by CVST and cannot, on their own, demonstrate that CVST elevates affective-symptom risk above the perinatal baseline.

## Data Availability

The raw data supporting the conclusions of this article will be made available by the authors, without undue reservation.
