# Supplementary material for: Anxiety and depression after pregnancy-related cerebral venous sinus thrombosis: associations with functional disability at discharge
Source: Front Public Health. 2026 Jun 12;14:1833585. doi: 10.3389/fpubh.2026.1833585 (PMC13303033; doi:10.3389/fpubh.2026.1833585)
Supplement: Supplementary file 1 [file Data_Sheet_1.docx]

# Supplementary Material

# Contents

Table S1. Glasgow Coma Scale (GCS) — scoring rubric.

Table S2. Modified Rankin Scale (mRS) — scoring rubric.

Table S3. Hamilton Anxiety Rating Scale (HAMA) — item scoring.

Table S4. 24-item Hamilton Depression Rating Scale (HAMD-24) — item scoring.

Table S5. Linear regression of HAMA / HAMD-24 on discharge mRS (unadjusted, minimally adjusted, fully adjusted, with HC3 robust SE).

Table S6. Stratified analysis by perinatal phase with formal interaction test.

Table S7. Antepartum sensitivity analysis adding obstetric outcome as a covariate.

Table S8. Spearman rank correlations between discharge mRS and 3-month affective scores (overall and by phase).

Table S9. Regression diagnostics for the fully adjusted models.

Figure S1. Spearman scatter plot of mRS vs HAMA / HAMD-24.

Figure S2. Directed acyclic graph (DAG) used to guide covariate selection.

Figure S3. Forest plot of stratified β estimates by perinatal phase (also referenced as Figure 3 in the main text).

## Supplementary Table S1. Glasgow Coma Scale (GCS)

| Component | Description | Score |
| --- | --- | --- |
| Eye Opening | Spontaneous / To speech / To pain / None | 4 / 3 / 2 / 1 |
| Verbal Response | Oriented / Confused / Inappropriate / Incomprehensible / None | 5 / 4 / 3 / 2 / 1 |
| Motor Response | Obeys / Localizes pain / Withdraws / Flexion / Extension / None | 6 / 5 / 4 / 3 / 2 / 1 |
| Total Score | Sum of all components | 3–15 |

Severity classification: Mild 13–15; Moderate 9–12; Severe ≤8.

## Supplementary Table S2. Modified Rankin Scale (mRS)

| Score | Functional Description |
| --- | --- |
| 0 | No symptoms |
| 1 | No significant disability |
| 2 | Slight disability |
| 3 | Moderate disability |
| 4 | Moderately severe disability |
| 5 | Severe disability |
| 6 | Death |

Outcome grouping in this study: Good 0–2; Poor 3–6.

## Supplementary Table S3. Hamilton Anxiety Rating Scale (HAMA), 14 items

| Item No. | Item Description | Score Range |
| --- | --- | --- |
| 1 | Anxious mood | 0–4 |
| 2 | Tension | 0–4 |
| 3 | Fears | 0–4 |
| 4 | Insomnia | 0–4 |
| 5 | Intellectual (cognitive) symptoms | 0–4 |
| 6 | Depressed mood | 0–4 |
| 7 | Somatic (muscular) symptoms | 0–4 |
| 8 | Somatic (sensory) symptoms | 0–4 |
| 9 | Cardiovascular symptoms | 0–4 |
| 10 | Respiratory symptoms | 0–4 |
| 11 | Gastrointestinal symptoms | 0–4 |
| 12 | Genitourinary symptoms | 0–4 |
| 13 | Autonomic symptoms | 0–4 |
| 14 | Behavior at interview | 0–4 |

Total range 0–56. Severity classification: 0–7 (no/minimal); 8–14 (mild); 15–23 (moderate); ≥24 (severe).

## Supplementary Table S4. 24-item Hamilton Depression Rating Scale (HAMD-24)

| Item No. | Item Description | Score Range |
| --- | --- | --- |
| 1 | Depressed mood | 0–4 |
| 2 | Feelings of guilt | 0–4 |
| 3 | Suicide | 0–4 |
| 4 | Insomnia (early) | 0–2 |
| 5 | Insomnia (middle) | 0–2 |
| 6 | Insomnia (late) | 0–2 |
| 7 | Work and activities | 0–4 |
| 8 | Psychomotor retardation | 0–4 |
| 9 | Agitation | 0–4 |
| 10 | Psychic anxiety | 0–4 |
| 11 | Somatic anxiety | 0–4 |
| 12 | Gastrointestinal somatic symptoms | 0–2 |
| 13 | General somatic symptoms | 0–2 |
| 14 | Genital symptoms | 0–2 |
| 15 | Hypochondriasis | 0–4 |
| 16 | Loss of weight | 0–2 |
| 17 | Insight | 0–2 |
| 18 | Diurnal variation | 0–2 |
| 19 | Depersonalization/derealization | 0–4 |
| 20 | Paranoid symptoms | 0–4 |
| 21 | Obsessive and compulsive symptoms | 0–4 |
| 22 | Helplessness | 0–4 |
| 23 | Hopelessness | 0–4 |
| 24 | Worthlessness | 0–4 |

Total range 0–76. Severity classification: 0–7 (no/minimal); 8–16 (mild); 17–23 (moderate); ≥24 (severe).

## Supplementary Table S5. Linear regression of HAMA and HAMD-24 on discharge mRS

| Outcome | Model | β (95% CI) | p (model) | p (HC3 robust) |
| --- | --- | --- | --- | --- |
| HAMA | Unadjusted | 8.52 (7.61, 9.42) | <0.001 | <0.001 |
| HAMA | Minimally adjusted | 8.45 (7.13, 9.76) | <0.001 | <0.001 |
| HAMA | Fully adjusted | 8.37 (7.02, 9.72) | <0.001 | <0.001 |
| HAMD-24 | Unadjusted | 8.41 (7.39, 9.42) | <0.001 | <0.001 |
| HAMD-24 | Minimally adjusted | 7.42 (5.97, 8.86) | <0.001 | <0.001 |
| HAMD-24 | Fully adjusted | 7.25 (5.78, 8.72) | <0.001 | <0.001 |

β denotes the change in symptom score per one-point increase in mRS at discharge. Minimally adjusted models include age, GCS category at admission, and any cerebral haemorrhage and/or infarction. Fully adjusted models additionally include perinatal phase, any autoimmune disease, and anaemia. Heteroscedasticity-consistent (HC3) robust standard errors were computed following the small-sample recommendation of Long and Ervin (Long JS, Ervin LH. Using heteroscedasticity-consistent standard errors in the linear regression model. The American Statistician 2000;54(3):217–24).

## Supplementary Table S6. Stratified analysis by perinatal phase, with formal interaction test

| Outcome | Subgroup | n | β (95% CI) | Interaction p |
| --- | --- | --- | --- | --- |
| HAMA | Antepartum | 43 | 7.01 (4.75, 9.28) | 0.075 |
| HAMA | Postpartum | 72 | 9.34 (7.62, 11.06) |  |
| HAMD-24 | Antepartum | 43 | 5.23 (2.81, 7.66) | 0.897 |
| HAMD-24 | Postpartum | 72 | 8.17 (6.31, 10.02) |  |

Each stratum-specific model was minimally adjusted (age, GCS category, any haemorrhage/infarction). The interaction p value is from the cross-product term mRS × perinatal phase fitted in a single overall model with the same covariates.

## Supplementary Table S7. Antepartum sensitivity analysis adding obstetric outcome

| Outcome | Term | β (95% CI) | p |
| --- | --- | --- | --- |
| HAMA | mRS at discharge | 6.97 (4.62, 9.32) | <0.001 |
| HAMA | Mid-trimester induction (vs abortion) | −0.99 (−5.93, 3.95) | 0.687 |
| HAMA | Live birth (vs abortion) | 0.06 (−5.90, 6.02) | 0.983 |
| HAMD-24 | mRS at discharge | 5.19 (2.67, 7.71) | <0.001 |
| HAMD-24 | Mid-trimester induction (vs abortion) | −0.17 (−5.46, 5.12) | 0.948 |
| HAMD-24 | Live birth (vs abortion) | 0.71 (−5.68, 7.09) | 0.823 |

Models fit within the antepartum subgroup only (n = 43; 42 with non-missing HAMA/HAMD), additionally adjusted for age, GCS category, and any haemorrhage/infarction. Reference category for delivery mode is artificial abortion. The mRS coefficient is virtually unchanged from the stratified estimate without delivery_mode (HAMA 7.01; HAMD-24 5.23), indicating that obstetric outcome does not mediate the disability–affect relationship.

## Supplementary Table S8. Spearman rank correlations between discharge mRS and 3-month affective scores

| Pair | Subgroup | ρ | n | p |
| --- | --- | --- | --- | --- |
| Discharge mRS vs HAMA | Overall | 0.752 | 111 | <0.001 |
| Discharge mRS vs HAMA | Antepartum | 0.779 | 42 | <0.001 |
| Discharge mRS vs HAMA | Postpartum | 0.712 | 69 | <0.001 |
| Discharge mRS vs HAMD-24 | Overall | 0.680 | 111 | <0.001 |
| Discharge mRS vs HAMD-24 | Antepartum | 0.626 | 42 | <0.001 |
| Discharge mRS vs HAMD-24 | Postpartum | 0.699 | 69 | <0.001 |

ρ = Spearman rank correlation coefficient. Sample size differs from 115 because four patients died before the 3-month assessment (HAMA/HAMD missing).

## Supplementary Table S9. Regression diagnostics for the fully adjusted models

| Model | Test | Statistic | p value | Interpretation |
| --- | --- | --- | --- | --- |
| HAMA, fully adjusted | Breusch–Pagan | 9.20 | 0.326 | Homoscedastic |
| HAMA, fully adjusted | Shapiro–Wilk | 0.959 | 0.002 | Mild non-normality |
| HAMD-24, fully adjusted | Breusch–Pagan | 7.46 | 0.488 | Homoscedastic |
| HAMD-24, fully adjusted | Shapiro–Wilk | 0.988 | 0.450 | Approx. normal |

Breusch–Pagan tests heteroscedasticity (low p indicates non-constant variance). Shapiro–Wilk tests residual normality (low p indicates departure from normality). The combination of acceptable Breusch–Pagan results, mild non-normality in HAMA only, and concordant Spearman estimates supports reporting linear-model results with HC3 robust standard errors as the primary inferential summary.

## Supplementary Figure S1. Spearman correlations between modified Rankin Scale at discharge and 3-month affective symptoms

##
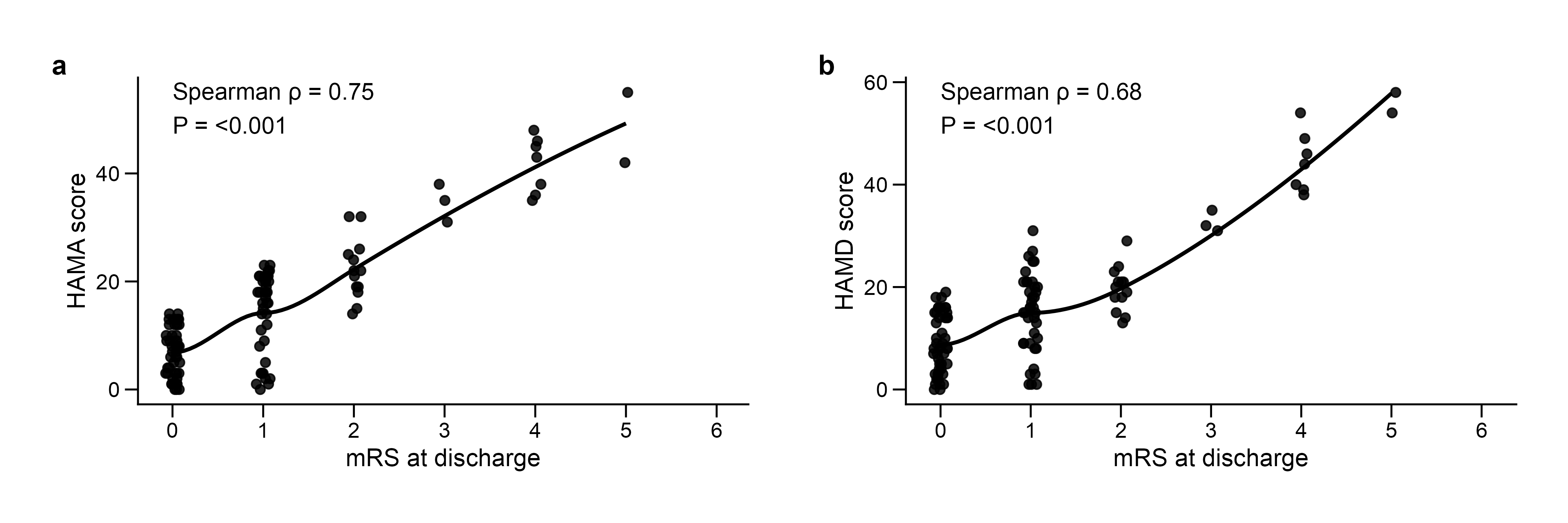


Side-by-side scatter plots (panel a, HAMA; panel b, HAMD-24). Each dot represents one patient; the solid line is a locally weighted (loess) smoother. Spearman correlation coefficients (ρ) and p values are annotated in each panel.

## Supplementary Figure S2. Directed acyclic graph (DAG) used to guide covariate selection

##
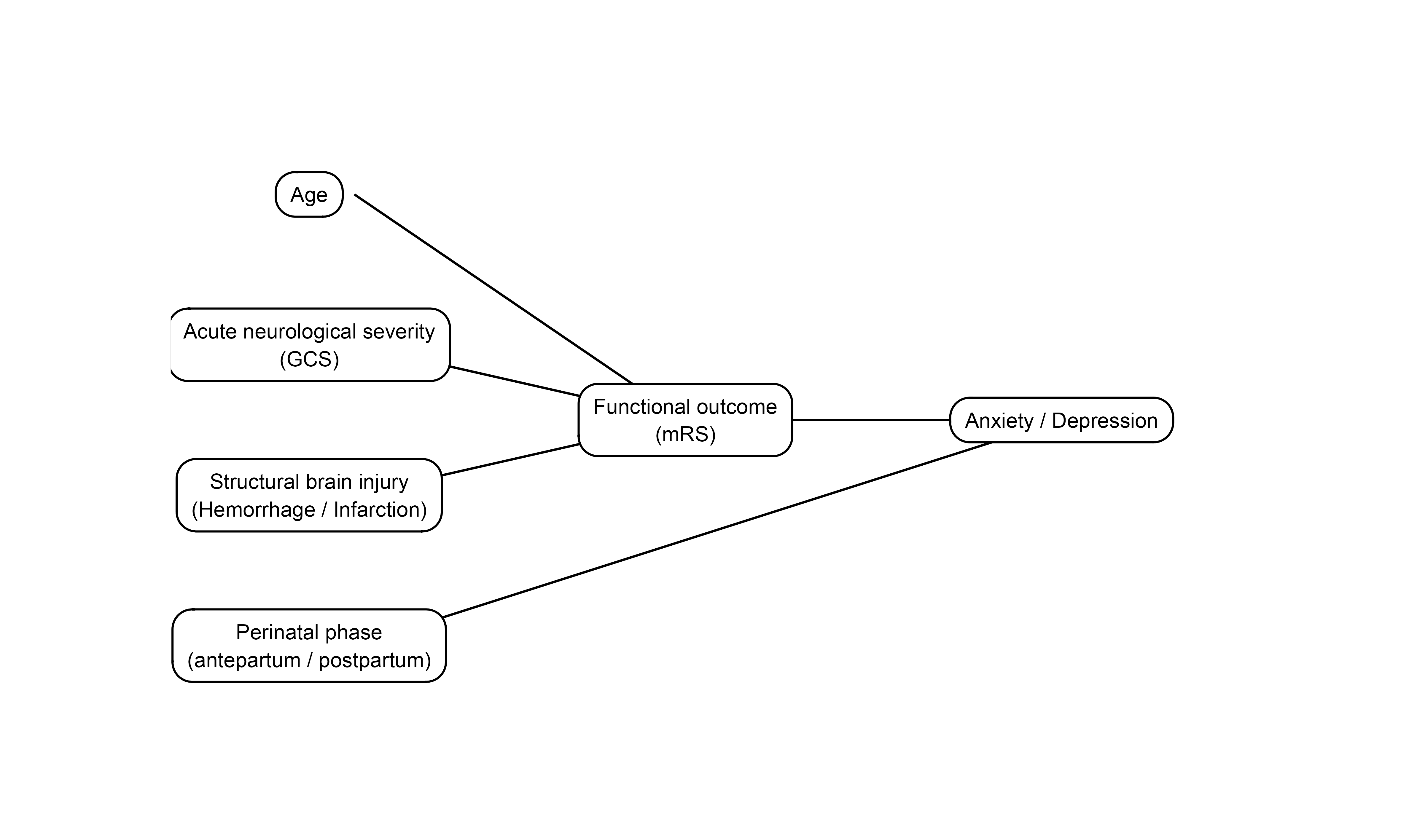


Nodes: Age, acute neurological severity (Glasgow Coma Scale), structural brain injury (haemorrhage and/or infarction), and perinatal phase (antepartum vs postpartum) are upstream variables; functional outcome (mRS at discharge) is the exposure; anxiety and depression at 3 months constitute the outcome. The minimally adjusted model blocks backdoor paths through age, GCS category, and brain injury; the fully adjusted model additionally adjusts for perinatal phase and key comorbidities.

Source TIFF: FigS2_DAG_with_perinatal.tiff (200 mm × 120 mm; 600 dpi).

## Supplementary Figure S3. Forest plot of stratified β estimates by perinatal phase

**
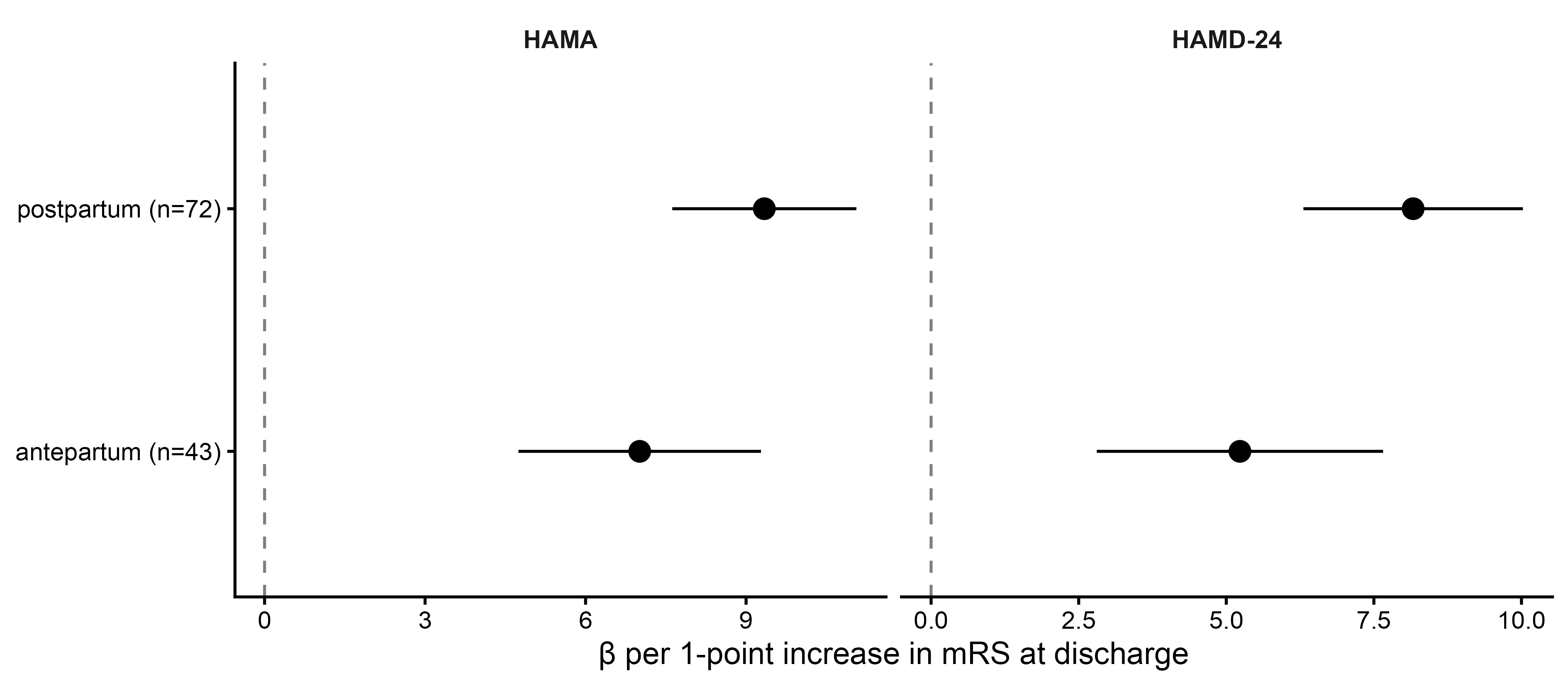
**

Point estimates and 95% confidence intervals for the regression coefficient of mRS at discharge, fitted separately within antepartum-onset (n = 43) and postpartum-onset (n = 72) subgroups, with the same minimally adjusted covariate set. Each panel shows one outcome (HAMA on the left, HAMD-24 on the right). The vertical dashed line at zero indicates no association. This same figure is also referenced as Figure 3 in the main text.

# Supplementary references

Long JS, Ervin LH. Using heteroscedasticity-consistent standard errors in the linear regression model. The American Statistician 2000;54(3):217–224.
